# Rejection Capacity of Nanofiltration Membranes for Nickel, Copper, Silver and Palladium at Various Oxidation States

**DOI:** 10.3390/membranes11090653

**Published:** 2021-08-26

**Authors:** Brooms Thabo, Bamidele Joseph Okoli, Sekomeng Johannes Modise, Simphiwe Nelana

**Affiliations:** 1 Chemistry Department, Faculty of Applied and Computer Sciences, Vaal University of Technology, Private Bag X021, Vanderbijlpark 1911, South Africa; brooms@vut.ac.za (B.T.); joe@vut.ac.za (S.J.M.); simphiwen@vut.ac.za (S.N.); 2 Department of Chemical Sciences, Faculty of Science and Technology, Bingham University, Karu PMB 005, Nasarawa State, Nigeria

**Keywords:** nanofiltration, membranes, precious metals, oxidation states, pH, rejection, wastewater

## Abstract

Electroplating and metalworking industries produce enormous amounts of waste containing heavy metals in their effluents, leading to potential threats to biotic and abiotic life. According to regulation, heavy metal contamination must be kept within the regulated standard of a few parts per million, which has led to a recent pique in interest in the utilization of nanofiltration technology for metal recovery. The effect of feed pH, pressure, metal concentration, and oxidation of metal on the rejection of heavy metal ions using three commercial nanofiltration membranes (NF, NF90, and NF270) were explored. To begin, studies of electrolyte salts, contact angle, and water permeability were employed to characterize the nanofiltration membranes. A dead-end module was used to test the permeation and retention capacities of the nanofiltration membranes. The results showed an increase in salt rejection for all metals examined irrespective of the membrane, at a pH below the isoelectric point. For divalent cations, the NF90 membrane achieved recovery capacities of 97% and 85% at 200 ppm and 20 ppm respectively, as compared to the recovery observed for Ni^2+^, Cu^2+^, and Pd^2+^ ions by NF and NF270. At a pH 2, 20 ppm and 5 bar, the NF90 membrane had the highest percent recovery, but at a pH 3, the recovery was at 95%. Mono and divalent stable Ag+ and Ni^2+^ ions showed a comparatively high percent recovery as compared to Pd^2+^ and Cu^2+^, which have high molecular weight and charge effect. In the presence of chelating agents, the membrane surface area is increased, resulting in high divalent ion recovery capacities due to favourable interaction with the polyamide functional group of the membranes. This study establishes the significance of oxidation in high removal efficiency cation in varying experimental conditions.

## 1. Introduction

Metal pollution is becoming increasingly problematic to the environment due to metallurgical and mining processes. Such industrial processes generate a large quantity of wastewater contaminated with a variety of heavy metals. Treatment of wastewater to meet the environmental standard has become a necessity in the wake of sustainable development goals and the circular economy [1]. Over the years, several processes, such as solvent extraction, distillation, evaporation, ion-exchange, chemical oxidation, chemical precipitation, and flotation have been developed and employed for the recovery and separation of metal in the industry [2]. Nanofiltration membranes have grown in popularity in recent years in the chemical, petrochemical, biotech, and desalination industries, since the nanofiltration membrane technology removes operational problems that plague older techniques [3,4]. Nanofiltration membranes are synthetic polymers with charged groups that may be used to separate charged metals from water. In terms of separation properties, nanofiltration is a pressure-driven membrane separation technology that sits in between reverse osmosis (RO) and ultrafiltration (UF). The advantages of nanofiltration polymeric membranes are that they come with the application at low operation pressure, they have a high permeate flux, and they have a high retention of multivalent salt ions. Nanofiltration membrane performance is influenced by a number of factors, including the chemical composition of the membrane active layer, the aqueous ion speciation, pressure effects, concentration of ions, pH levels, membrane charge, and membrane pore size [5,6]. 

**Figure 1:**
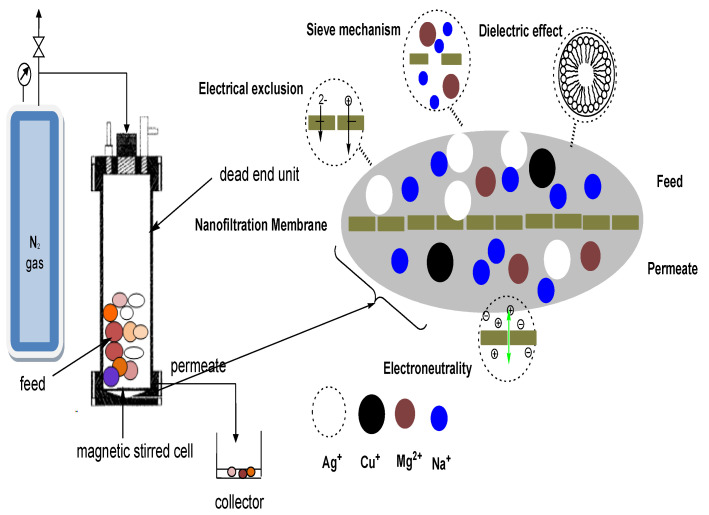
Nanofiltration separation mechanism.

In addition, solution diffusion, sieving, the Donnan effect, dielectric exclusion, and electromigration all occur in nanofiltration, making it appropriate for the separation of both charged and uncharged solutes (Figure 1). In a number of successful studies, nanofiltration membranes have been used as heavy metal removal devices. 

The nature of the membrane surface charge and pore size as well as the type of dissolved metal species can all be affected by the feed pH, and therefore, the membrane separation efficiency. The impact of feed pH on the removal of certain heavy metals and flow permeate using nanofiltration membranes have previously been investigated by López et al. [7], Ramos et al. [8], and Siddique et al. [9]. On the other hand, the examination of multiple heavy metals with varying oxidation states on commercial membranes under the same conditions provides a wealth of information about the membranes and their applicability under various situations. Mined mineral resources like palladium (Pd), silver (Ag), copper (Cu), and nickel (Ni) are useful both economically and industrially; however, they are extremely harmful when discharged without treatment [10]. Since the advent of membrane technology (MT), it has received a lot of attention in the separation and recovery of precious metal from industrial and environmental wastewater [11,12]. Specifically, in South Africa, nanofiltration membranes have proven to be a viable option for water, pharmaceutical, biochemical, mining, and wastewater treatment application [13,14]. 

According to López [15], polyamide-based nanofiltration membranes have shown their potential for treating acidic mine waters containing relatively high concentrations of transition elements (e.g., Fe, Al, Cu, and Zn) and, in some cases, rare earth elements of total concentrations of about 80 mM, in addition to moderate concentrations of sulphuric acid. Polyamide-based nanofiltration membranes allow the recovery of sulphuric acid to permeate and concentrate the metals and rare earth elements in the feed tank solution. Unfortunately, the industries have implemented a “take-make, consume, and dispose of” pattern of growth. This linear model is based on the assumption that raw materials are abundant and available, easy to obtain, and cheap to dispose of. Circular economy systems maintain the added value in products for as long as possible, while the generation of waste is avoided or reduced [16]. 

Because the properties of commercially available flat sheet nanofiltration membranes vary, understanding the rejection behaviour for a given membrane–metal ion problem is crucial for the evaluation of a nanofiltration treatment strategy. The aim of this study was to examine the performance of three commercial nanofiltration membranes under a variety of experimental conditions and oxidation states in order to better understand the relationship between solute rejection, pressure, and feed pH to guide in the selection of an ideal membrane for a specific process.

## 2. Materials and Methods

Three flat sheets (FS) of nanofiltration membranes purchased from Dow/Filmtec (Gauteng, South Africa) were studied, namely NF90, NF270, and NF. NF90 and NF270 are made from polyamide (productional functional group, (WantItAll (Pty) Ltd, Gauteng, South Africa)) surface material bearing a negative charge [17,18]. NF, which is a polypiperazine-amide, is also made from the same monomer as the latter two mentioned membranes (Table 1). All membranes were collected from an unwounded spiral wound model. A solution mixture with a concentration of 20 to 100 ppm was exposed to a nanofiltration membrane at trans-membrane pressure ranging as follows; 5, 10, 15, and 20 bar, respectively. 

### 2.1. Chemical Analysis

Divalent metal salts (CuCl_2_, NiCl_2_, and PdCl_2_) and monovalent metal salts (AgCl and CuCl) (Sigma-Aldrich, Freising, Germany) were prepared as mining synthetic samples for the rejection of cation species with concentrations ranging from 5 to 200 ppm at room temperature. All of the model solutions were prepared with deionised water. The stainless-steel unit has a circular flat sheet cell with two halves fastened together for tightening the bolts, which was used in the experiment for retention of metal species in the solution. Nitrogen gas (N_2_) was used as the driving force throughout the whole experiment. Scanning electron microscopy (SEM) (JSM-7610F, Freising, Germany) was applied for membrane morphology for both virgin and used flat sheet elements. This was to visualise the surface material of the membrane and concentration polarisation occurrence on the polymeric material. Shimadzu Graphite Furnace Atomizer 7000 atomic absorption spectroscopy (Duisburg, Germany) was used to determine the metal contents’ concentration present in feed, retentive and filtrate dispersion (permeate). As for adjustments of solution to acidity, 0.1M 37% HCl was used, and the pH of the solution was determined using the Crimson pH meter (Alella, Barcelona). Metrohm 712 conductometer (Woodmead, Sandton, South Africa) was utilised for quantification of dissolved ionic metal species.

### 2.2. Experimental Set Up (Dead-End Module)

**Figure 2:**
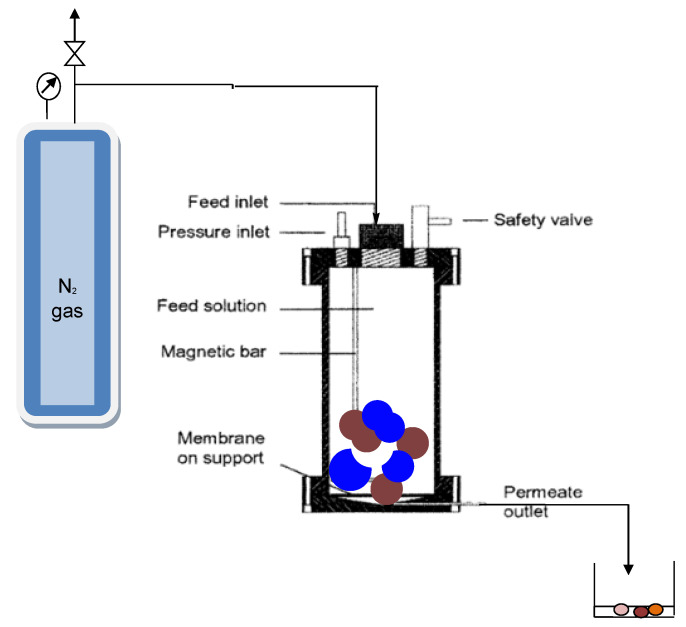
Dead-end unit module used for this study.

Permeation and retention analyses were performed on 1 L capacity dead-end module as the bench-scale unit operated at pressures of 25 bar with nitrogen gas (Figure 2). The unit was fitted with a Teflon-coated magnetic stirrer supported on the upper lid by a steel rod. Stirring was required to homogenize the sample and to minimize concentration polarization [12]. Disc samples of the different membranes with a diameter of 9 cm and an effective area of 6.36 × 10^−4^ m^2^ were cut and placed on a porous support disc. The hold-up volume underneath the porous support disc was ~1 mL. Permeate was collected from a Teflon tube into a measuring cylinder. The unit can be operated at pressure ranging from 5 to 20 bar for nanofiltration membranes’ specifications [19]. Solutions were constantly stirred at 500 rpm to homogenize the feed samples. The first 20 mL of permeate collected was discarded. Thereafter, 10 mL of permeate was collected at a specified time.

### 2.3. Characterization of the Materials

#### Water Permeability Study

Flat sheet membranes were characterized in terms of water permeability, solute permeation, and rejection capacity metals on the membranes’ material. Clean water permeability was determined for all three membranes, followed by permeation behaviour of charged solutes/electrolytes (NaCl and MgCl_2_) ions. 

For the clean water experiment, permeate flux, which is the volumetric rate of flow through the unit membrane area, can be expressed by Equation (1):

(1) $$J_{W}\frac{V}{S\cdot t}$$

If the feed and the retentate contains pure water, i.e., the osmotic pressure difference across the membrane becomes zero, the Darcy equation will be reduced to Equation (2):

(2) $$J_{W}=A_{w}\Delta P$$

An alternative approach for expressing water flux through the membrane is by using the following Hagen–Poiseuille Equation (3):

(3) $$J_{W}=\frac{\varepsilon \cdot r^{2}}{8\cdot \eta \cdot \tau }\cdot \frac{\Delta P}{\Delta x}$$

The tortuosity of the membrane can be approximated by Equation (4):

(4) $$\varepsilon \approx \frac{1}{\tau }$$

If both Darcy and Hagen–Poiseuille (Equations (2) and (3)) are combined, then Equation (5) for quantifying water permeability becomes:

(5) $$A_{W}=\frac{\varepsilon }{\tau }\cdot \frac{r^{2}}{8\cdot \eta \cdot \Delta x}$$

The value of A_w_ can experimentally be obtained from the slope of the plot of volume flux (L·m^−2^·h^−1^·bar^−1^) versus pressure (bar). The viscosity of pure water is usually 0.001 Pa·s, and the membrane thickness (∆x) is taken as 1µm [20]. 

The procedure used for quantifications were directly adopted from the instrument manual. The observed rejection of salt is defined in Equation (6):

(6) $$R=\left(1-\frac{C_{p}}{C_{f}}\right)100$$

The nomenclature of the common meanings of the symbols and subscripts used in Equations (1)–(6) are listed in Table 2:

### 2.4. Scanning Electron Microscopy (SEM)

**Figure 3:**
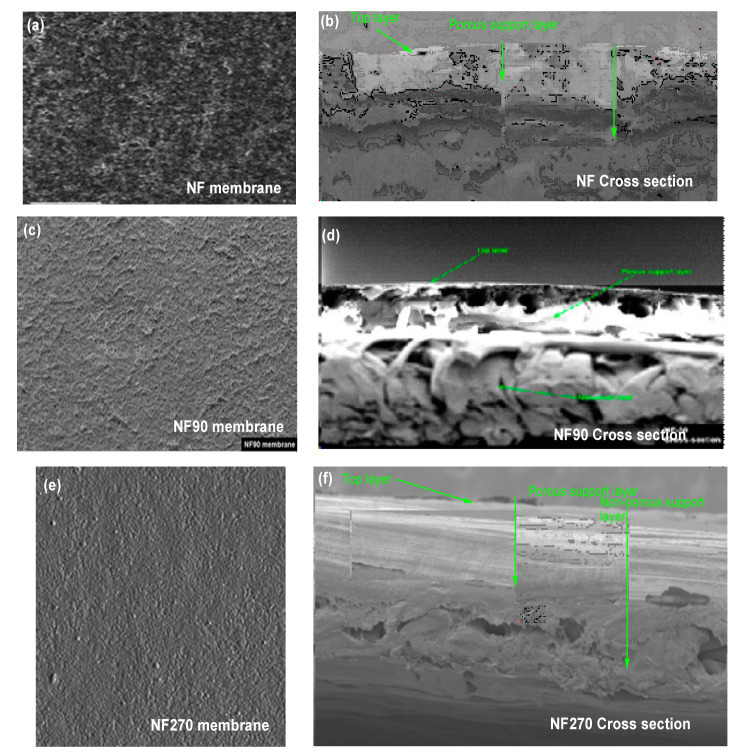
(**a**) NF Top view, (**b**) cross sectional view of NF, (**c**) NF90 Top view, (**d**) cross sectional view of NF90, (**e**) NF270 Top view and (**f**) cross sectional view of NF90.

The thin film composite membrane (NF90) was characterized by scanning electron microscopy (JSM-7610F, Freising, Germany) to obtain visual information of the pore size and surface properties. The membrane samples’ surface was trimmed down to 0.5 × 0.5 cm, then mounted to a sample plate (brass disk) and sputter-coated with gold. After that, a scanning electron microscope (low vacuum) was used to photograph the sample at a magnification of 5000 times. The cross-section of the membranes indicates the three-polymer layer (top layer, porous support layer, and non-woven layer) produced from amides monomers. The top layer is the most important layer, where the charge of the membrane lies based on the functional group as a preservative of the producer or manufacture. As indicated below, Figure 3 (a) displays the NF Top view, (b) displays the cross-sectional view of NF, (c) displays the NF90 Top view, (d) displays the cross-sectional view of NF90, (e) displays the NF270 Top view, and (f) displays the cross-sectional view of NF90. The cross-section micrography proposed a homogeneous morphology. Hence, fouling materials may hinder the layer surface during the filtration cycle. 

### 2.5. Contact Angle Measurement 

The contact angles of NF, NF90, and NF270 were measured using a DSA10-MK2 contact angle analyzer (BmbH Co., Bremerhaven, Germany). The sessile drop method was used to measure the contact angles of deionized water (3 L) on the dried surfaces of the membranes at an ambient temperature of 25 °C. The drop had a 5 L capacity. The contact angles were established using images taken 5 s after the drop was introduced. At least 10 measurements on different parts of the membrane were taken and averaged to provide the contact angle of the various membranes. The data presented indicated an average of six measurements ± standard deviation.

## 3. Results and Discussion

NF270 has a very thin semi-aromatic piperazine-based polyamide active layer, while NF90 consists of a fully aromatic polyamide active layer. NF90s’ pore radius is smaller than that of NF270, and again, their structures are slightly different despite having the polyamide thin film composite (TFC) [21,22]. Yildirim et al. [23] reported that the NF membrane has slightly higher permeate flux as compared to NF270 during trans-membrane pressure (TMC) at different time intervals during salt test removal.

All three commercial membranes bare a negatively charged surface (top layer) as an active layer due to the amide functional group (Table 1). 

Confirmation of the water flux results are inconsistent with the suppliers’ specification of N270 having the largest pore radius sizes and highest levels of flux permeate as compared to NF90 and NF. Although the two membranes NF90 and NF270 are produced and prepared from the same monomer, their performance slightly differs as indicated by the water permeability results (Table 3). However, a very dense polyamide material (NF90) indicated the lowest flexibility as compared to NF and NF270. 

**Figure 4:**
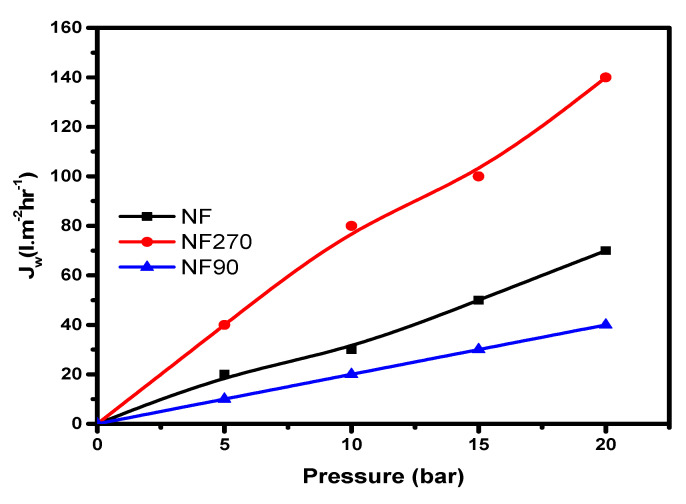
Clean water flux on all three membranes (dead-end module).

According to the specification for flux ranges by Ochando et al. [24], nanofiltration flux permeability values are between 1–12 (L·m^−2^·h^−1^·bar^−1^), with a pressure range between 5–20 bar. All of the membranes tested fall within the specification ranges stated above, which implies that they are nanofiltration membranes (Figure 4). 

### 3.1. Membrane Wettability 

Table 4 shows the contact angle of NF, NF90, and NF270 membranes. NF270 was more permeable than NF90, but had poorer solute rejection efficiency. The hydrophobicity or hydrophilicity of the membranes’ surface is shown by the contact angle.

The contact angle of all three membranes is below 90°, hence, all the investigated membranes have hydrophilic surface. In comparison, the contact angle of NF90 was the greatest, followed by NF and NF270 in that order, indicating that NF90 had a more hydrophobic surface.

### 3.2. Salt Retention Measurements

#### 3.2.1. Single Salt Rejection

**Figure 5:**
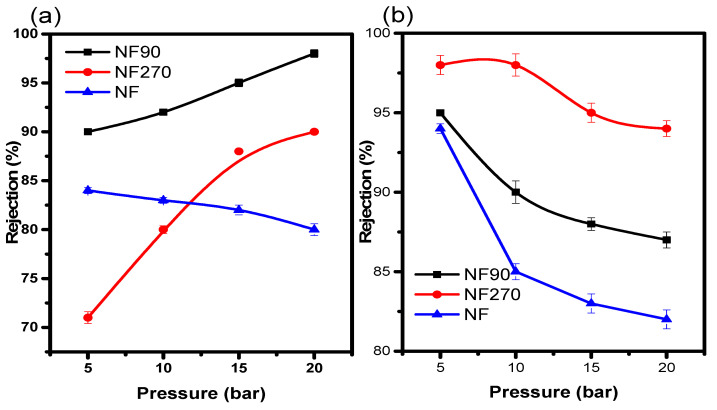
(**a**) Na^+^ and (**b**) Mg^2+^ rejection at pH 2.0 and 20 ppm.

In a membrane separation method, it is crucial for the membranes to possess a satisfactory salt rejection. Salt rejection by a nanofiltration membrane is principally affected by the membrane properties, salt activities, and water chemistry. However, the elaborate mechanisms for salt rejection are unclear. In this study, the behaviours of TFC membranes’ solute rejections (R) with different void (pore) size were evaluated. NF90 and NF270 had rejection capacities of 90% at the lowest pressure (Figure 5a). The results proved that both cations had good rejection behaviour on the respective membranes at pH 2.0. As expected, NF90 with the smallest void size gave the highest rejection capacity for Na^+^. Comparatively, Na^+^ (a monovalent cation) had a lower rejection capacity on all three membranes as compared to Mg^2+^ (a divalent cation). This was due to the fact that it had the highest molecular weight of an element as a compound, and also due to the electrostatic interaction between the membrane surface and solution (Figure 5b).

Principally, this interaction is governed by the Donnan exclusion, which is caused by the electrostatic interactions of ionic solutes with fixed electric charges attached to the membrane matrix, which is called the “charge effects”. However, there are two other popular mechanisms that may also explain salt rejection in nanofiltration membrane; the dielectric exclusion and the hydration mechanism. 

Furthermore, the enhanced Na^+^ rejection in NF270 and NF90 at high pressure is most likely due to an increase in membrane efficiency at this pressure, which is consistent with reports on the efficiency of NF90 at low pressure by Emamjomeh et al. [25] and Van der Bruggen and Vandecasteele [26]. The low efficiency of NF270 and NF90 at low pressure is more likely due to the increase in membrane surface charge than the pores at low pH. When the membrane surface became more negatively charged than the pores, with increasing pH, the decrease in rejection became more obvious in the Mg^2+^ ion.

#### 3.2.2. Metal Rejection Capacity 

**Figure 6:**
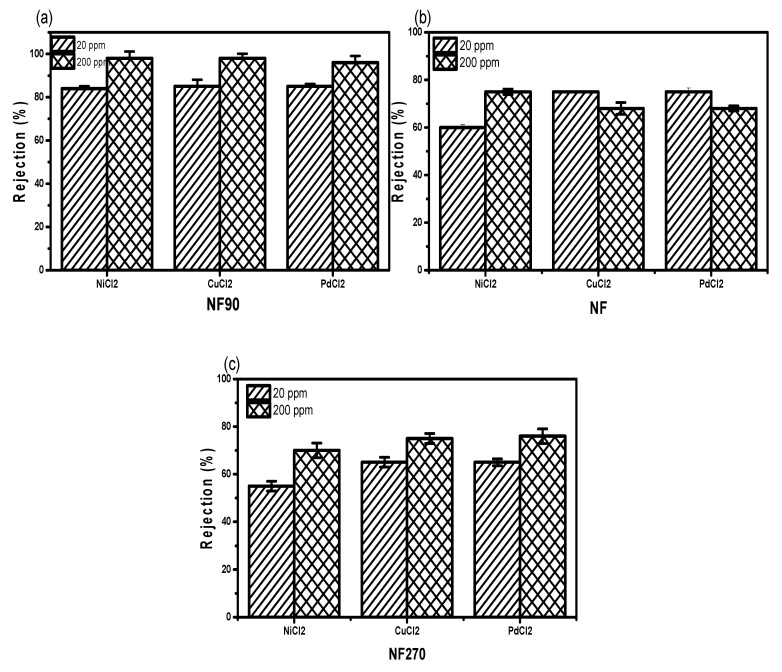
Divalent cation rejection capacities for (**a**) NF90, (**b**) NF, and (**c**) NF270 at two different concentrations.

NF90—which was considered to be the membrane with the smallest pores as compared to NF and NF270—was favoured for its high metal rejection potential as a negatively charged membrane in a single ion solution. At 20 ppm, the NF90 membrane gives the highest rejection than all investigated concentrations (Figure 6a–c). 

This rejection capacity has to do with membrane surface conditions as well as the concentration level of the salts and molecular weight cut-off for the membrane. However, NF270 has the lowest rejection capacity at 200 ppm because of the high molecular weight cut-off of 200. The low percent rejection observed for NF and NF270 as compared to NF90 was complemented by high surface charge (Figure 6b,c). Due to their sparingly stable conditions, Ni^2+^ and Cu^2+^ were slightly higher than Pd^2+^, which was not expected. By implication, the rejection capacity of the metal cation is not only dependent on concentration, molecular weight, and pH, but also on stability of the ion (Figure 6).

**Figure 7:**
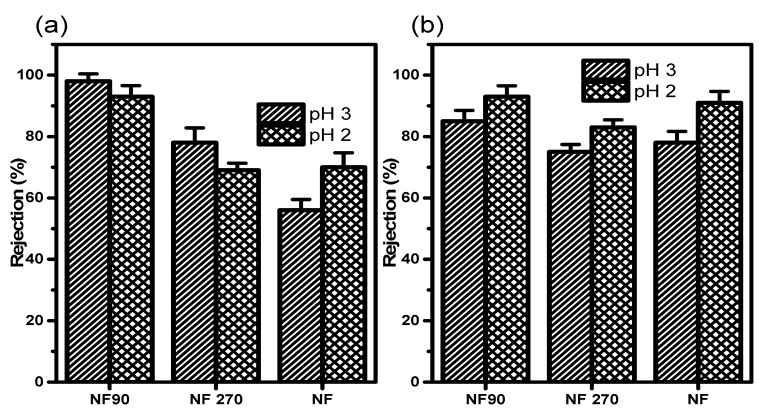
Rejection of (**a**) Cu^+^ in a 200 ppm CuCl solution at 5 bar and (**b**) Ag^+^ in a 200 ppm AgCl solution at 5 bar.

In Figure 7, the retention of CuCl and AgCl as a single solution mixture at 200 ppm gave a recovery of R ≈ 93 for Cu^+^ at 5 bar, thus favouring NF90 (dense polyamide material) amongst selected membrane elements at pH 3, compared to pH 2 as a monovalent ion. 

According to the manufacturers’ specifications, all selected membranes are negatively charged with respect to the functional group; however, the charge surface was modified to positive using HCl. Consequently, the lower retention observed for NF and NF270 was expected and in agreement with the observation that monovalent salt tends to be more rejected (lower molecular weight) as compared to bivalent (highest charge effect) and multivalent ions, which tend to have the highest recovery. These have to do with the membrane pore size on the nanosize surface material of the membrane. AgCl is stable compared to other silver compounds. Provided it is not in contact with UV light or being exposed to air [17], AgC1 had the highest recovery of 93% on NF90 at pH 3.0. According to the procedure described by Hussain et al. [27], NF90 and NF have isoelectric points at about pH 4.2 and 5.8 respectively. These indicate that the membrane has a zero or neutral charge, at the point of minimum ion rejection and maximum fluxed. This has to do with the membrane electroneutrality at those points [28]. 

**Figure 8:**
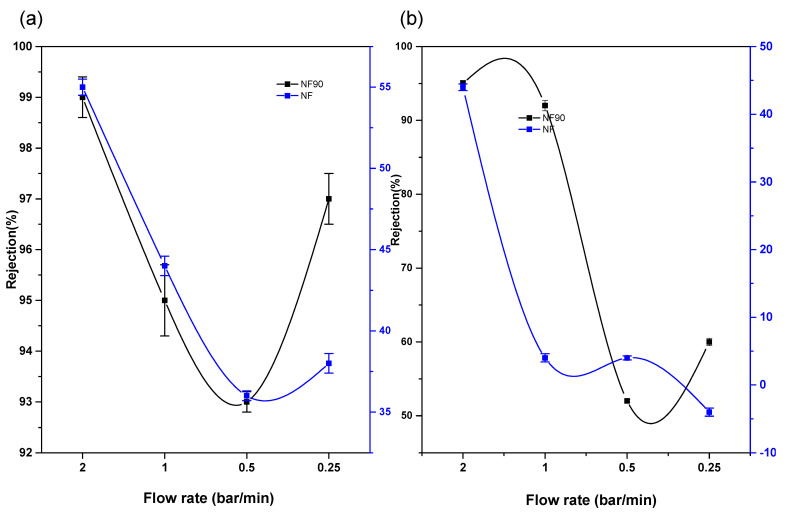
Membrane performance at 20 ppm and pH 2 (**a**) in the presence of chelating agent on Pd in Pd/Cu/Ni/EDTA mixture (**b**) without chelating agent on Pd in Pd/Cu/Ni/EDTA mixture.

**Figure 9:**
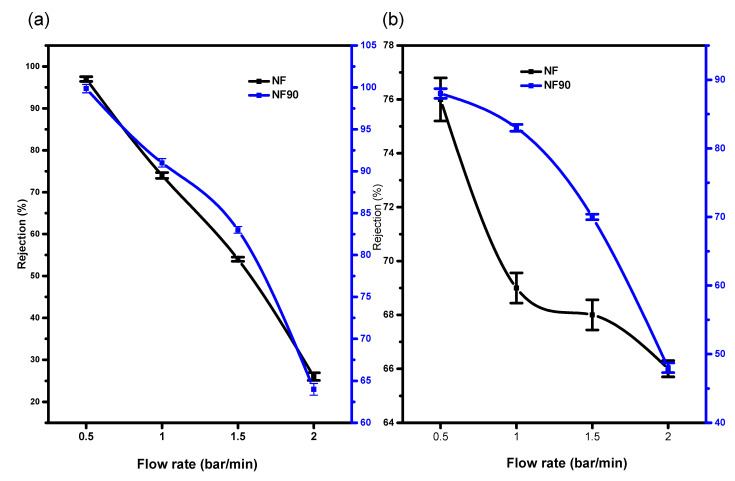
(**a**) Percentage rejection of Ni^2+^ (**b**) Cu^2+^ binding to (Na_2_EDTA) on Cu/Ni/EDTA at 20 ppm and pH 2 with chelating agent.

Figure 8 and Figure 9 indicate the retention potential of a membrane in the presence or absence of a chelating agent (Na_2_EDTA). Considering the effect of chelate on the salt retention of NF and NF90, the properties of NF and NF270 in this study have been somehow similar. Chelating agents have the potential for increasing the surface area of the membrane material [29]. The results indicated high recovery capacities due to binding or inactivation of the mobile cation. Hence, palladium, copper, and nickel chloride metal salts showed favourable interaction with the polyamide functional group of the membranes as compared to the interaction in the absence of the chelating agents. Both Cu^2+^ and Pd^2+^ had higher rejection capacities of R > 99% as compared to Ni^2+^ in the binary and multivalent cation mixtures at a minimum pressure of 5 bar. Consequently, the electrostatic interaction between the membrane material and the cation solution mixture had the highest charge density, making it possible for compounds with the highest molecular weight cut-off to be retained even more. 

The highest retention with or without the chelating agent for all membranes was observed at 2 bars per minute. The presence of the chelating agent in the complex mixture did not cause concentration polarization on the membrane top layer as suspected.

**Figure 10:**
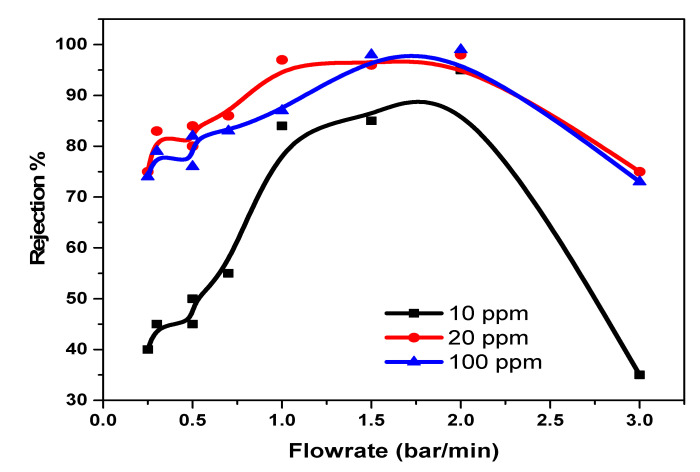
NF90 application on Pd^2+^ on multivalent mixture at various concentration.

The rejection capacities of Pd^2+^ in a multi-element mixture were also considered with the view of monitoring movement of the ions and understanding the rejection mechanism for optimal application. A combination of a mixture of Pd, Cu, and Ni was studied on the NF90 membrane. The result indicated an increase in the rejection capacity of Pd^2+^, with an increase in the pressure and concentration of the multi-element mixture at pH 3.0. In this case, the rejection of Pd^2+^ is influenced by the Donnan exclusions’ multiple charged co-ions, which has a greater rejection rate than single-charged co-ions. Therefore, negatively charged membranes are especially suitable for the separation of Pd^2+^ in a multi-element mixture. However, the reduction in the amount of cation recovered in the absence of the chelate is due to the fact that the recovery mechanism is solely dependent on solution diffusion, sieving, dielectric exclusion, and electromigration only. The optimal condition for Pd^2+^ removal is at a flowrate of 1.5 bar/min for 10 ppm, 20 ppm, and 100 ppm with rejection capacities of 83%, 95%, and 96% respectively. As for Figure 10, negative rejection for prolonged duration was observed. The membrane pore size was enlarged, thus increasing flux permeate. At the lowest trans-pressure, the highest recovery is achieved, which implies a low and cost-effective process and technique. 

## 4. Conclusions

Among the flat sheet elements tested, NF90 and NF membranes had the highest rejection capacity as compared to NF270, due to the latter’s large pore sizes. This was confirmed during the water permeability study on the characterization of the membranes. Solute retention (Na^+^) had a lower rejection capacity level as compared to Mg^2+^, the molecular compound with the highest molecular weight. This was due to its’ high retention of the membrane top layer (membrane charge lies), and also due to the electrostatic interaction between the membrane surface material and the solution. Pd^2+^, because of its higher molecular weight compared to Cu^2+^ and Ni^2+^, was highly rejected as a single solution mixture. Cu^2+^ has a very stable condition as compared to nickel and palladium. This had to do with the nature and physical properties during the chemical reaction process. NF90 and NF had the highest recovery, though they differ in pore size and molecular weight cut-off. This study has proven that retention of metals does not only rely on concentration and pressure, but also on the influence of pH and pressure existence. Both high and low concentration solution mixtures were considered, and pH 3.0 was found to be the most suitable range as compared to pH 2.0. Pd seemed to be the highest recovered cation in almost all of the ranges compared in the research.

The primary aim of this study is to recover these selected transition metals in artificial mining wastewater, as part of the economical beneficiation and empowering job creation around South Africa and in other parts of the continent using nanofiltration membranes. According to the findings in the study, solute retention (Na^+^) had a lower rejection capacity level as compared to Mg^2+^, the molecular compound with a higher molecular weight. Mg^2+^ was highly retained on the membrane top layer due to the electrostatic interaction between the membrane surface material and the solution. Moreover, the rejection of divalent ions is inconsistent with the findings of previous investigators. Furthermore, the introduction of chelating agents enhances the recovery capacities of the membranes with polarisation. The study has proven that while retention of Pd^2+^ in a multivalent mixture at various concentrations does rely on concentration and pressure, the metal rejection is also influenced by the pH of the existing solution.

## Figures and Tables

**Table 1:** Properties of different nanofiltration membranes and their suppliers.

Membrane	NF90	NF270	NF
Manufactures	Dow/Filmtec	Dow/Filmtec	Dow/Filmtec
Material	Polyamide	Polyamide	Polypiperazine
Membrane type	FS	FS	FS
Maximum operating temp. (°C)	45	45	45
Surface charge @ pH 7	−10 ± 2	−18 ± 1	−24 ± 2
pH range	2–11	2–11	3–10
MWCO (Da)	150	200	150

MWCO = Molecular weight cut-off, FS = Flat sheet, °C = Degree Celsius.

**Table 2:** Symbols and Nomenclature.

Symbols	Parameter	Units
V	Volume flux	L
S	Surface area of the membrane	m^2^
T	Time	h
J_w_	Permeate flux	L·m^−2^·h^−1^
∆P	Trans-membrane pressure	bar
A_w_	Water permeability coefficient	L·m^−2^·h^−1^·bar^−1^
Δπ	Osmotic pressure difference	bar
ε	Surface porosity	
τ	Membrane tortuosity	
η	Viscosity	Pa·s
Δx	Membrane thickness	m
r	Pore radius	
c_p_	Concentration of solute on the permeate side	mg/L
c_f_	Concentration of solute on the feed side	mg/L
R	Observed rejection of salt	%

**Table 3:** Clean water permeability value for nanofiltration membranes (dead-end module).

Membrane	A_w_ (L·m^−2^·h^−1^·bar^−1^)
NF90	2.10
NF	3.58
NF270	7.46

**Table 4:** Contact angle for investigated nanofiltration membranes.

Membrane	Contact Angle (°)
NF90	47.3 ± 2.0
NF	32.1 ± 2.3
NF270	26.1 ± 2.1

## Data Availability

Not applicable.
